# Biliverdin Reductase-A Deficiency Brighten and Sensitize Biliverdin-binding Chromoproteins

**DOI:** 10.1247/csf.20010

**Published:** 2020-06-25

**Authors:** Kenju Kobachi, Sota Kuno, Shinya Sato, Kenta Sumiyama, Michiyuki Matsuda, Kenta Terai

**Affiliations:** 1 Laboratory of Bioimaging and Cell Signaling, Research Center for Dynamic Living Systems, Graduate School of Biostudies, Kyoto University; 2 Department of Molecular and Cellular Physiology, Graduate School of Medicine, Kyoto University; 3 Laboratory for Mouse Genetic Engineering, RIKEN Center for Biosystems Dynamics Research; 4 Department of Pathology and Biology of Diseases, Graduate School of Medicine, Kyoto University

**Keywords:** *in vivo* imaging, near-infrared fluorescent protein, biliverdin, biliverdin reductase, optogenetic tool

## Abstract

Tissue absorbance, light scattering, and autofluorescence are significantly lower in the near-infrared (NIR) range than in the visible range. Because of these advantages, NIR fluorescent proteins (FPs) are in high demand for *in vivo* imaging. Nevertheless, application of NIR FPs such as iRFP is still limited due to their dimness in mammalian cells. In contrast to GFP and its variants, iRFP requires biliverdin (BV) as a chromophore. The dimness of iRFP is at least partly due to rapid reduction of BV by biliverdin reductase-A (BLVRA). Here, we established *biliverdin reductase-a* knockout (*Blvra^–/–^*) mice to increase the intracellular BV concentration and, thereby, to enhance iRFP fluorescence intensity. As anticipated, iRFP fluorescence intensity was significantly increased in all examined tissues of *Blvra^–/–^* mice. Similarly, the genetically encoded calcium indicator NIR-GECO1, which is engineered based on another NIR FP, mIFP, exhibited a marked increase in fluorescence intensity in mouse embryonic fibroblasts derived from *Blvra^–/–^* mice. We expanded this approach to an NIR light-sensing optogenetic tool, the BphP1-PpsR2 system, which also requires BV as a chromophore. Again, deletion of the *Blvra* gene markedly enhanced the light response in HeLa cells. These results indicate that the *Blvra^–/–^* mouse is a versatile tool for the *in vivo* application of NIR FPs and NIR light-sensing optogenetic tools.

## Introduction

For fluorescence microscopy of mammalian tissues, near-infrared (NIR) light is preferable to visible light, because of its low absorbance, scattering, and tissue autofluorescence (Konig, 2000; Weissleder and Ntziachristos, 2003). Accordingly, in the last 10 years, several research groups have been developing genetically encoded NIR fluorescent proteins (FPs) such as iRFPs (Filonov *et al.*, 2011; Shcherbakova and Verkhusha, 2013), miRFPs (Shcherbakova *et al.*, 2016), IFPs (Shu *et al.*, 2009; Yu *et al.*, 2014), mIFPs (Yu *et al.*, 2015), and Wi-Phy (Auldridge *et al.*, 2012) from bacterial phytochrome photoreceptors (BphPs) (Chernov *et al.*, 2017). These NIR FPs are frequently used for multicolor imaging (Donnelly *et al.*, 2017; Janssen *et al.*, 2017; Maryu *et al.*, 2016; Weinhard *et al.*, 2018) and for spectral separation with optogenetic tools driven by visible light (Qian *et al.*, 2019; Shcherbakova *et al.*, 2018).

BphP-derived FPs require biliverdin IXα (BV) as a chromophore (Filonov *et al.*, 2011). BV is the intermediate product of mammalian heme metabolism and is ubiquitously present in mammalian tissues (Tran *et al.*, 2014). The most widely used NIR FP, iRFPs (Filonov *et al.*, 2011; Shcherbakova *et al.*, 2016), binds endogenous BV more efficiently than do other NIR FPs, such as IFPs (Shu *et al.*, 2009; Yu *et al.*, 2015, 2014) and smURFP (Rodriguez *et al.*, 2016). Nevertheless, at least in some cell types, endogenous BV is insufficient for maximal iRFPs intensity (Filonov *et al.*, 2011; Piatkevich *et al.*, 2017; Vasavda *et al.*, 2019). Therefore, it is expected that the full potential of iRFPs can be achieved by increasing the intracellular BV level.

BV supplementation promotes the fluorescence of NIR FPs, including iRFPs, in mammalian cells to some extent (Filonov *et al.*, 2011; Piatkevich *et al.*, 2017; Shemetov *et al.*, 2017); however, due to the low membrane permeability, the enhancement is usually modest. Biliverdin dimethyl ester (BVMe_2_), a membrane-permeable BV derivative, greatly enhances the smURFP signal (Rodriguez *et al.*, 2016). However, BVMe_2_ is hardly applicable to iRFPs due to its inefficient affinity to BphP-based NIR FPs (Shemetov *et al.*, 2017). Furthermore, BVMe_2_ is rapidly cleaved by esterases and degraded in blood (Rodriguez *et al.*, 2016), which hampers *in vivo* application. In fact, the efficacy of intravenous BV administration in intravital imaging varies among studies: BV administration was shown to increase the fluorescence level of IFP1.4 in the liver (Shu *et al.*, 2009), but not the fluorescence of smURFP in HT1080 tumor xenografts (Rodriguez *et al.*, 2016).

An approach to elevate the BV level is co-expression of heme oxygenase (HO), which catalyzes the reduction of heme to produce BV. Co-expression of HO increases the fluorescence intensity of several NIR FPs in mammalian cells (Rodriguez *et al.*, 2016; Shemetov *et al.*, 2017; Yu *et al.*, 2014) and in *Drosophila* (Yu *et al.*, 2015). However, enhancement of the brightness in HeLa cells is limited to less than 25%, in contrast to the nearly 100% increase by BV supplementation (Shemetov *et al.*, 2017).

In mammalian cells, BV is reduced to bilirubin IXa by two isoforms of biliverdin reductase (BLVR), BLVRA and BLVRB (O’Brien *et al.*, 2015). BLVRA is ubiquitously expressed in adult tissues and is responsible for the reduction of biliverdin IXα to bilirubin IXα (Komuro *et al.*, 1996; Yamaguchi *et al.*, 1994). Meanwhile, BLVRB is expressed in the fetal tissue and responsible for the reduction of biliverdin IXβ to bilirubin IXβ (Yamaguchi *et al.*, 1994), which is not detected in the adult stage.

In the current study, we sought to increase the BV level by an alternative strategy: blocking the BV reduction. Therefore, we generated *Blvra^–/–^* mice. Knockout animals were born with Mendelian ratios and did not exhibit any major abnormality, except for BV accumulation and concomitant bilirubin reduction in the gallbladder. Intravital imaging from *Blvra^–/–^* mice showed improved iRFP intensity in all tested tissues. Furthermore, the signal-to-noise ratios of NIR light-sensing optogenetic tools were also increased in BLVRA-deficient HeLa cells.

## Materials and Methods

### Animals

Mice were housed in a specific pathogen-free facility in temperature-controlled rooms with a 14 h light/10 h dark cycle, and received a routine chow diet and water *ad libitum*. The animal protocols were reviewed and approved by the Animal Care and Use Committee of the Kyoto University Graduate School of Medicine (No. 10584).

The *Blvra^–/–^* mice were developed by a CRISPR/Cas9 system targeting the murine *Blvra* gene (NM_026678.4). Two gRNAs, 5'-ACCTGGACACATATCCAATC-3' and 5'-CGAGAAATGCCACTGAACGC-3', were co-injected with Cas9 mRNA into cytoplasm of the fertilized eggs obtained from C57BL/6N mice (Sunagawa *et al.*, 2016). Initial screening by direct sequencing identified three F0 mice with either small insertions, deletions, or a large DNA-fragment inversion between the two gRNA-target sites. We selected an F0 mouse containing a large inversion in one *Blvra* allele for further analysis. The F0 mouse was crossed with B6N-Tyrc-Brd/BrdCrCrl mice, also known as B6 albino mice (Japan SLC, Hamamatsu, Japan). The offspring mice, *Blvra^+/–^*, were further crossed to obtain *Blvra^–/–^* B6 albino mice.

NuCyM mice have been reported previously (Imanishi *et al.*, 2018). In these mice, the morphological marker NuCyM is expressed throughout the body, on a B6 albino background. NuCyM^+/–^ *Blvra^–/–^* mice were obtained by crossing *Blvra^–/–^* and NuCyM^+/–^ mice. For imaging, 7- to 9-week-old male or female mice were used.

### Mouse genotyping

Genomic DNAs were extracted from mouse tails by using SimplePrep (TaKaRa Bio, Kusatsu, Japan) and used for genotyping PCR. Primer sets were designed for the *Blvra* wild-type (WT) allele (5'-TGGTAGTGGTTGGTGTTGGCC-3' and 5'-CCACTACTCGGCATGGTTCT-3') and the inverted allele (5'-TCATATATTGATCTTCTTTTCGGTT-3' and 5'-CCACTACTCGGCATGGTTCT-3'), yielding 216 and 226 bp products, respectively.

### Plasmids

cDNAs of iRFPs were the kind gifts of Dr. Vladislav Verhusha (Shcherbakova and Verkhusha, 2013). pCSIIbleo-iRFP713-P2A-EGFP and pCSIIbleo-iRFP670-P2A-EGFP contain the cDNA of iRFP713 or iRFP670, followed by the coding sequence of the self-cleaving P2A peptide (Kim *et al.*, 2011) and the EGFP cDNA. cDNAs of RpBphP1-mCherry, PpsR2-mVenus-CAAX, and NIR-GECO1 were obtained from Addgene (Watertown, MA; #79832, #79835, #113680) and inserted into lentivirus expression vectors, generating pCSIIbsr-RpBphP1-mCherry, pCSIIhyg-PpsR2-mVenus-CAAX, and pCSIIbsr-NIR-GECO1-P2A-EGFP, respectively. The envelope plasmid pCMV-VSV-G-RSV-Rev was a kind gift from Dr. Hiroyuki Miyoshi (RIKEN BioResource Center). The cDNA of the SV40 large T antigen (Suzuki *et al.*, 2004) was inserted into the retroviral vector pMSCV-pac (Hawley *et al.*, 1994), generating pMSCV-pac-LT. Another envelope plasmid, pMD2.G, was purchased from Addgene (#12259). pGP was provided by Dr. Tsuyoshi Akagi (Akagi *et al.*, 2003).

### Reagents

Biliverdin IXα was purchased from Cayman Chemical (Ann Arbor, MI), dissolved in DMSO at a final concentration of 25 mM, and stored at –20°C. Ionomycin was purchased from Calbiochem (San Diego, CA), dissolved in DMSO at a final concentration of 20 mM, and stored at –20°C.

### Cell culture and establishment of stable cell lines

Lenti-X 293T cells were obtained from TaKaRa Bio (Kusatsu, Japan). The BLVRA KO HeLa cells were kind gifts of Dr. Kazuhiro Aoki (Uda *et al.*, 2017). Mouse embryonic fibroblasts (MEFs) were isolated from E14.5 embryos. Lenti-X 293T cells and MEFs were cultured in D-MEM (high-glucose; FUJIFILM Wako Pure Chemical, Osaka, Japan) supplemented with 10% (v/v) fetal bovine serum (Sigma-Aldrich, St. Louis, MO) and 1% (v/v) penicillin/streptomycin (Nacalai Tesque, Kyoto, Japan) at 37°C in a humidified 5% CO_2_/95% air atmosphere. MEF cells were immortalized by infection with SV40 large T antigen-expressing retrovirus and subsequent selection in the presence of 2 μg mL^–1^ puromycin (InvivoGen, San Diego, CA). For the preparation of cells stably expressing iRFP713-P2A-EGFP or other ectopic proteins, a lentiviral expression system was employed: pCSII-based lentiviral vector, psPAX2, and pCMV-VSV-G-RSV-Rev were co-transfected into Lenti-X 293T cells with polyethylenimine (Polyscience Inc., Warrington, PA). Transfected cells were then selected in medium containing either 100 μg mL^−1^ Zeocin (InvivoGen) or 10 μg mL^−1^ blasticidin S (FUJIFILM Wako Pure Chemical).

### Fluorescence imaging of tissue culture cells

Confocal fluorescence images of MEFs were acquired with an IX81 inverted microscope (Olympus, Tokyo, Japan) equipped with an FV1000 confocal imaging system (Olympus) and a 20×/0.75 NA dry objective lens (UPLSAPO 20X; Olympus) under the following sets of conditions. For GFP imaging, we used a 488 nm excitation laser, DM 405/488/559/635 dichroic mirror, and 510–560 nm range for detection; for iRFP670, we used a 635 nm excitation laser, DM 405/488/559/635 dichroic mirror, and 660–760 nm for detection; for iRFP713, we used a 635 nm excitation laser, DM 405/488/559/635 dichroic mirror, and 680–780 nm range for detection; for mVenus, we used a 488 nm excitation laser, DM 405/488/559/635 dichroic mirror, and 510–610 nm range for detection; for mCherry, we used a 559 nm excitation laser, DM 405/488/559/635 dichroic mirror, and 575–650 nm range for detection; for NIR-GECO1, we used a 635 nm excitation laser, DM 405/488/559/635 dichroic mirror, and 655–755 nm range for detection. The microscope was controlled by FV10-ASW software (Olympus). An external 735 nm LED illuminator (Optocode, Tokyo, Japan) was used as shown in Fig. 5. Images were processed and analyzed with MetaMorph software (Molecular Devices, San Jose, CA).

For transient expression of RpBphP1-mCherry and PpsR2-mVenus-CAAX, HeLa cells were transfected with the expression vectors using 293fectin (Thermo Fisher Scientific, Waltham, MA). Cells were plated on glass-based 24-well plates or 35-mm dishes (AGC Inc., Tokyo, Japan) coated with collagen type I (Nitta Gelatin, Osaka, Japan). Before imaging, the cells were conditioned for more than 1 h in FluoroBrite DMEM (Thermo Fisher Scientific) supplemented with 1% (v/v) GlutaMAX (Thermo Fisher Scientific), 0.1% (w/v) bovine serum albumin, 20 mM 4-(2-hydroxyethyl)-1-piperazineethanesulfonic acid and 10% (v/v) fetal bovine serum (Sigma-Aldrich).

### Preparation of mice for intravital imaging

Mice were anesthetized with inhalation of 1.0% to 1.5% isoflurane (FUJIFILM Wako Pure Chemical) using a small animal anesthetizer (Biomachinery Chiba, Japan) and placed in either the prone or supine position on an electric heat pad maintained at 37°C. Ear skin imaging were performed as described previously (Hiratsuka *et al.*, 2015). For liver, kidney or pancreas imaging, each organ was pulled out of the abdominal cavity through an incision (Mizuno *et al.*, 2014) and immobilized with a custom-made vacuum-stabilized imaging window ((Sano *et al.*, 2016); Olympus Engineering, Tokyo, Japan). The suction force was set to approximately 20 kPa.

For brain imaging, we used a thinned-skull cranial window technique modified from a previous report (Yang *et al.*, 2010). Individual mice were anesthetized with 1.0% to 1.5% isoflurane. Body temperature was maintained at 37°C with a heating pad during surgery. After fixation of the head by ear bars, the scalp was removed. Then, 1% lidocaine (AstraZeneca, Cambridge, UK) solution was injected to the periosteum of the skull for topical anesthesia. The skull above the imaging area was thinned with a high-speed microdrill (Argofile, Tokyo, Japan) equipped with a round steel burr (0.7 mm diameter; GC Corporation, Tokyo, Japan) at 30,000 rpm. The thus-prepared window was sealed with a circular coverglass (3 mm diameter, #1; Matsunami, Osaka, Japan), a skull holder plate with a round hole was put onto the window and glued to the skull with cyanoacrylate glue (Toagosei, Tokyo, Japan), and the mouse was fixed to a custom-made skull immobilization stage via the skull holder plate.

### Two-photon excitation microscope (TPEM) and image processing

We used an FV1200MPE-BX61WI upright microscope (Olympus) equipped with a 25×/1.05 water-immersion objective lens (XLPLN25XWMP; Olympus), an InSight DeepSee Ultrafast laser (Spectra-Physics, Santa Clara, CA), a two-channel GaAsP detector unit, and two multi alkali detectors. The excitation wavelengths for mCherry and iRFP were 1040 nm and 1300 nm, respectively. Filters used for image acquisition were two dichroic mirrors, DM552 and DM649, and three emission filters: BA495-540 (Olympus) for the second harmonic generation (SHG), FF01-593/46 (Semrock, Rochester, NY) for mCherry and FF01-708/75 (Semrock) for iRFP. FV10-ASW (Olympus) was used to control the microscope and to acquire images. Z-sectioning of the brain was performed in 3 μm steps from the dura mater to a depth of 650 μm. Images were processed and analyzed with MetaMorph software or Fiji (https://imagej.net/Fiji).

### Preparation for splenocytes

Spleens were minced with scissors. A single-cell suspension was generated by passing through a ϕ40 μm cell strainer. The flow-through fraction was washed with PBS by centrifugation at 500 ×g for 5 min at 4°C. Red blood cells were removed by lysis with ACK lysing buffer (Thermo Fisher Scientific) and centrifugation at 500 ×g for 5 min at 4°C.

### Flow cytometry analysis

Cells suspended in PBS containing 3% (v/v) fetal bovine serum (Sigma-Aldrich) were analyzed with a FACS Aria IIIu cell sorter (Becton Dickinson, Franklin Lakes, NJ). The following combinations of lasers and emission filters were used for the detection of fluorescence: for the fluorescence of mCherry, a 561 nm laser and a 610DF20 filter (Omega Optical, Brattleboro, VT); for the fluorescence of iRFP713, a 633 nm laser and a 730AF45 filter (Omega Optical); for the fluorescence of 7-amino-actinomycin D (7-AAD), a 488 nm laser and a DF695/40 filter (Omega Optical). Cells were first gated for size and granularity to exclude cell debris and aggregates, and dead cells were excluded by 7-AAD (BD Pharmingen, San Diego, CA). Data analysis was performed using FlowJo software (Tree Star, Ashland, OR).

### Statistical analysis

All statistical analyses were performed on Excel (Microsoft, Redmond, WA). No statistical analysis was used to predetermine the sample size. Student’s *t*-test was used to evaluate statistically significant differences. *P* values less than 0.05 were considered statistically significant.

## Results

### Generation of Blvra^–/–^ mice

**Fig. 1:**
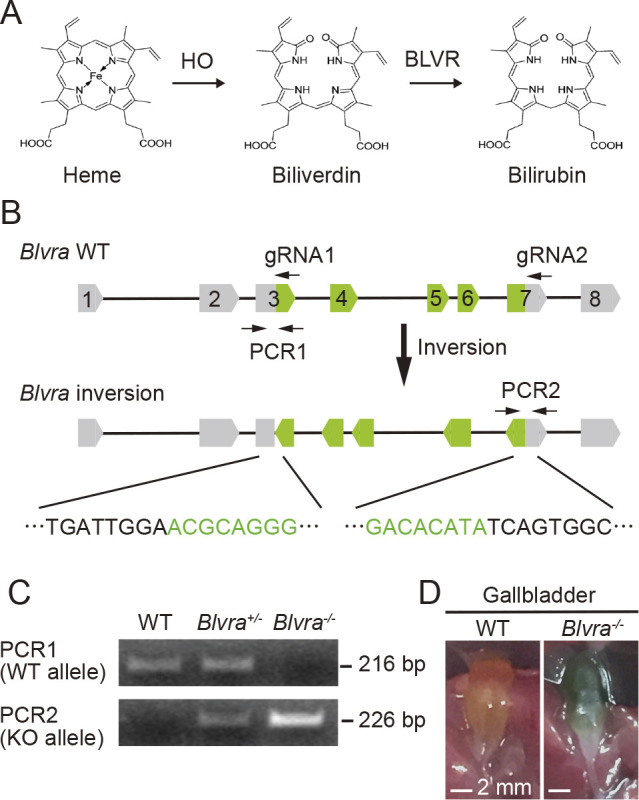
Generation of *Blvra* gene-deficient mice. (A) Heme reduction process. (B) Strategy for disrupting the mouse *Blvra* gene. Two gRNAs were used for CRISPR/Cas9-mediated mutagenesis. Positions of two pairs of PCR primers for the genotyping are also shown. (C) Genotyping PCR for WT and inverted alleles of the *Blvra* gene. Fragments of 216 and 226 bp indicate the WT and *Blvra*-deficient alleles, respectively. (D) The gallbladder in WT and *Blvra^–/–^* mice.

The emerging demand for intravital imaging in mammalian tissues has promoted the development of NIR FPs, which require BV as the chromophore. Although BV is generated from heme throughout the body (Fig. 1A), the concentration of endogenous BV is insufficient for maximal fluorescence brightness of NIR FPs (Filonov *et al.*, 2011; Piatkevich *et al.*, 2017; Vasavda *et al.*, 2019). Because BV is reduced primarily by BLVRA in mammalian cells (Fig. 1A) (McDonagh, 2001), we generated *Blvra^–/–^* mouse lines, hoping to achieve a higher BV concentration and thereby a greater brightness of NIR FPs. The knockout was performed by the CRISPR-Cas9 system (Doudna and Charpentier, 2014). Two gRNAs targeting exons 3 and 7 of the *Blvra* gene (Fig. 1B) were mixed and co-injected with Cas9 mRNA into fertilized mouse eggs. The mutations were confirmed by direct sequencing. Three out of 14 F0 mice carried mutations in *Blvra*. We chose a mouse line with an inverted mutation in *Blvra* (Fig. 1B), which guarantees complete elimination of the protein product. After several rounds of backcrossing and inbreeding, F4 offspring were genotyped by PCR. Two pairs of PCR primers were designed to selectively amplify the WT or the inverted allele (Fig. 1B). As anticipated, a 216 bp and/or a 226 bp fragment were detected to allow genotyping (Fig. 1C). The *Blvra^–/–^* mice were selected and maintained as a mouse line. The *Blvra^–/–^* offspring were born without detectable anomaly at least until the 11th generation. Consistent with previously generated *Blvra^–/–^* mice (Chen *et al.*, 2018), lack of the BLVR activity was apparently seen from the green gallbladder in our mice (Fig. 1D), indicating the accumulation of BV.

### Increased iRFPs fluorescence in Blvra^–/–^ MEF cells

**Fig. 2:**
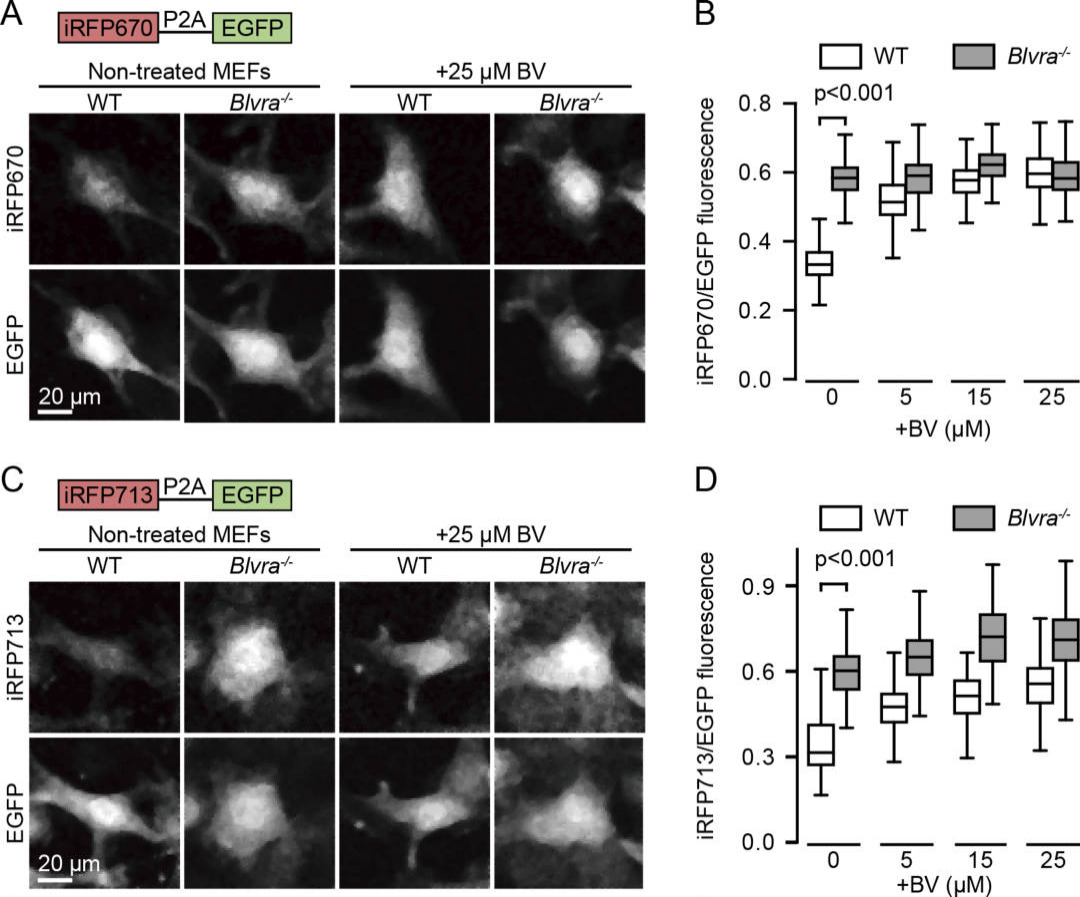
Increase in iRFPs brightness in *Blvra^–/–^* MEF cells. (A, C) Confocal images of WT and *Blvra^–/–^* MEF cells expressing iRFP670-P2A-EGFP (A) or iRFP713-P2A-EGFP (C). Before imaging, cells were precultured in medium with or without 25 μM BV for 36 h, and washed twice with PBS. (B, D) Normalized intensities of iRFP670 (B) and iRFP713 (D) with various exogenous BV concentrations. The iRFP intensities obtained from each cell were divided by those of EGFP for normalization. The edges on the box plots indicate the first and third quartiles, with the line in the middle being the median. The whiskers on the boxplots extend another 1.5× the interquartile range from the edges of the boxes, respectively. More than 150 cells from one or two imaging sessions were analyzed for each condition. P-values were obtained using two-tailed unpaired Student’s *t*-test.

Next, we validated the effects of *Blvra* gene knockout on the intensity of iRFPs by using MEF cells derived from the WT and *Blvra^–/–^* mice. MEFs were isolated and immortalized by the expression of SV40 large T antigen as described previously (Suzuki *et al.*, 2004). Stable cell lines for iRFP670 or iRFP713 were established using a lentiviral expression system. EGFP, which does not require an exogenous chromophore, was co-expressed via a P2A self-cleavable peptide and used as an internal control of the expression level; thus, the fluorescence intensity of iRFPs was evaluated as a ratio to that of EGFP. As expected, the intensity of iRFP670 fluorescence in *Blvra^–/–^* MEF cells was 1.7-fold brighter than that in WT MEF cells (Fig. 2A and B). The intensity in the WT was augmented to the level of *Blvra^–/–^* in the presence of exogenous BV (Fig. 2B, white boxes), indicating that iRFP670 expression was comparable between the two cell lines. In contrast, the BV supplementation did not increase the intensity in the *Blvra^–/–^* cells (Fig. 2B, gray boxes), suggesting the presence of sufficient endogenous BV. The *Blvra* knockout also enhanced the intensity of iRFP713 (Fig. 2C and D). In this case, the intensity augmentation with exogenous BV was detected even in the *Blvra^–/–^* cells (Fig. 2D, gray boxes). We also ruled out the BV autofluorescence in this analysis (Fig. S1). In addition, the intensity in the *Blvra^–/–^* cells in media without BV addition is higher than that in WT cells under 25 μM BV containing media. These results demonstrate that *Blvra* knockout is more effective to increase fluorescence of iRFPs than BV supplementation.

### Increased brightness of iRFP713 in Blvra^–/–^ mice

**Fig. 3:**
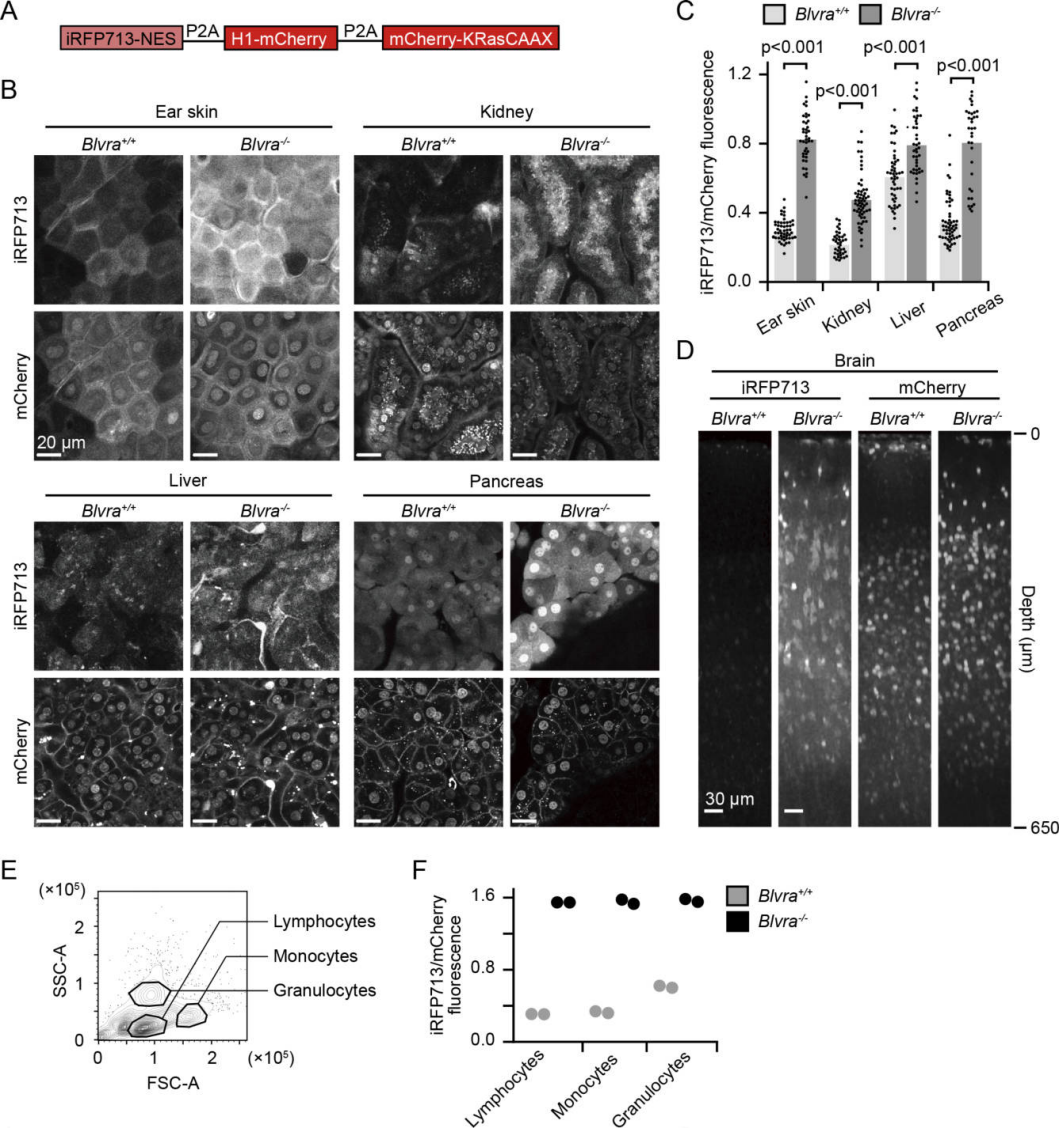
Increase in iRFP713 fluorescence in various organs of *Blvra^–/–^* mice. (A) Structure of a morphological marker, NuCyM. (B) Fluorescence images of the ear skin, kidney, liver, and pancreas in a NuCyM^+/–^ *Blvra^+/+^* or NuCyM^+/–^ *Blvra^–/–^* mouse. (C) Normalized intensities of iRFP713 in each tissue. iRFP713 intensities obtained from each cell were divided by those of mCherry for normalization. More than 34 cells from three or four independent mice were examined under each condition. Error bars represent SD. (D) XZ-plane view of the brain cortex. Images were reconstructed from Z-stack images with 3 μm steps. Similar results were obtained twice. (E) Representative counter plot of white blood cells after lysing red blood cells from *Blvra^+/+^* mouse. Cell populations were demarcated by forward (FSC) and side scatter (SSC). (F) Normalized intensities of iRFP713 in each cell population. After back-ground subtraction, mean iRFP713 intensities were normalized to mean mCherry intensity. Two mice were examined under each condition.

The increased iRFP fluorescence in MEFs encouraged us to proceed to *in vivo* imaging in *Blvra^–/–^* mice. For this purpose, we generated NuCyM^+/–^ *Blvra^–/–^* mice by crossing *Blvra^–/–^* mice with NuCyM mice, which ubiquitously express a set of morphological marker proteins throughout the body (Imanishi *et al.*, 2018). NuCyM consists of iRFP713 fused with a nuclear export signal (NES), mCherry fused with histone H1, and mCherry fused with the CAAX domain of K-Ras, all of which are cleaved off from a single peptide by means of the P2A self-cleavage peptides (Fig. 3A). The NuCyM^+/–^ *Blvra^–/–^* mouse was anesthetized and observed under a TPEM to acquire paired images of mCherry and iRFP713 (Fig. 3B). For the quantification, the fluorescence intensity of iRFP713 derived from a single cell was normalized to that of mCherry. The fluorescence intensities of iRFP713 in the ear skin, kidney, liver, and pancreas of NuCyM^+/–^ *Blvra^–/–^* mice were 2.8, 2.2, 1.3, and 2.3-fold higher than those in the WT mice (Fig. 3C). The variation among organs may suggest that BV exhibits a different catabolic efficiency in each organ. Of note, the increase in iRFP713 intensity by the *Blvra* deficiency was lowest in the liver, where secretion of BV and bilirubin to the bile duct actively occurs. Therefore, the extracellular transport machinery of BV may also affect the availability of BV. The different subcellular localization of iRFP713 among organs is probably due to incomplete cleavage of the P2A peptide (Imanishi *et al.*, 2018). To explore the application of *Blvra^–/–^* mice to neuroimaging, we observed the cerebral cortex of NuCyM^+/–^ *Blvra^–/–^* mice through an imaging window (Yang *et al.*, 2010). The fluorescence augmentation was more dramatic in the brain (2.4-fold to the NuCyM^+/–^ *Blvra^+/+^* mouse) and the iRFP713 signal was detected down to a depth of approximately 650 μm from the dura mater (Fig. 3D). Expression in the white blood cells in spleen was also examined by FACS (Fig. 3E). Similar to solid tissues, all white blood cells showed remarkable enhancement in iRFP713 intensity. A concern about *Blvra^–/–^* mice is that autofluorescence of BV may perturb the detection of NIR fluorescent proteins. However, we did not detect any difference in the fluorescence of iRFP713 channel between cells from *Blvra^–/–^* mice and *Blvra^+/+^* mice (Fig. S2). Taken together, these observations demonstrate that the *Blvra^–/–^* mouse line is a useful tool for *in vivo* imaging using NIR FPs.

### Increased brightness of NIR-GECO1 in Blvra^–/–^ MEF cells

**Fig. 4:**
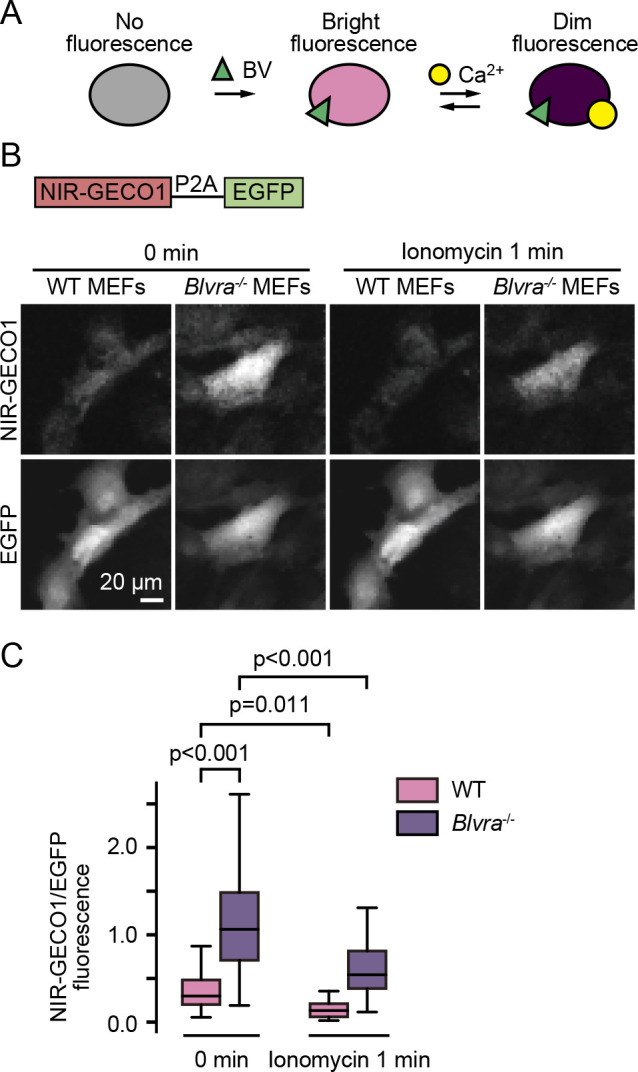
Increased fluorescence of NIR-GECO1 in *Blvra^–/–^* MEF cells. (A) Schematic representation of NIR-GECO1. (B) Confocal fluorescence images of WT and *Blvra^–/–^* MEF cells expressing NIR-GECO1. Images were obtained before and 1 min after 1 μM ionomycin addition. (C) Normalized intensities of NIR-GECO1. The NIR-GECO1 intensity obtained from each cell was divided by that of EGFP for normalization. The edges on the box plots indicate the first and third quartiles, with the line in the middle being the median. The whiskers on the boxplots extend another 1.5× the interquartile range from the edges of the boxes. The NIR-GECO1/EGFP ratio was quantified from more than 55 cells under each condition, in two independent experiments. P-values were obtained using two-tailed unpaired Student’s *t*-test.

We next evaluated the applicability of *Blvra^–/–^* mice to the calcium imaging. For this purpose, we used NIR-GECO1, a genetically encoded calcium ion indicator engineered based on mIFP (Qian *et al.*, 2019) (Fig. 4A). First, the intracellular NIR-GECO1 intensity was simply compared between the WT and *Blvra^–/–^* MEF cells. EGFP was used as an internal standard (Fig. 4B) as in Fig. 2. The *Blvra^–/–^* MEF cells showed 4.3-fold brighter NIR-GECO1 signals than the WT cells (Fig. 4B and C). Then, to eliminate the possible calcium level difference, the same set of cells was observed after adding ionomycin. NIR-GECO1 fluorescence was reduced by approximately 50% in both the WT and *Blvra^–/–^* MEF cells, confirming that the knockout merely augmented the available BV. These data suggest that BLVRA deficiency increases NIR-GECO1 intensity without perturbing the calcium response.

### Increased signal-to-noise ratio of the BphP1-PpsR2 optogenetic system in BLVRA KO HeLa cells

**Fig. 5:**
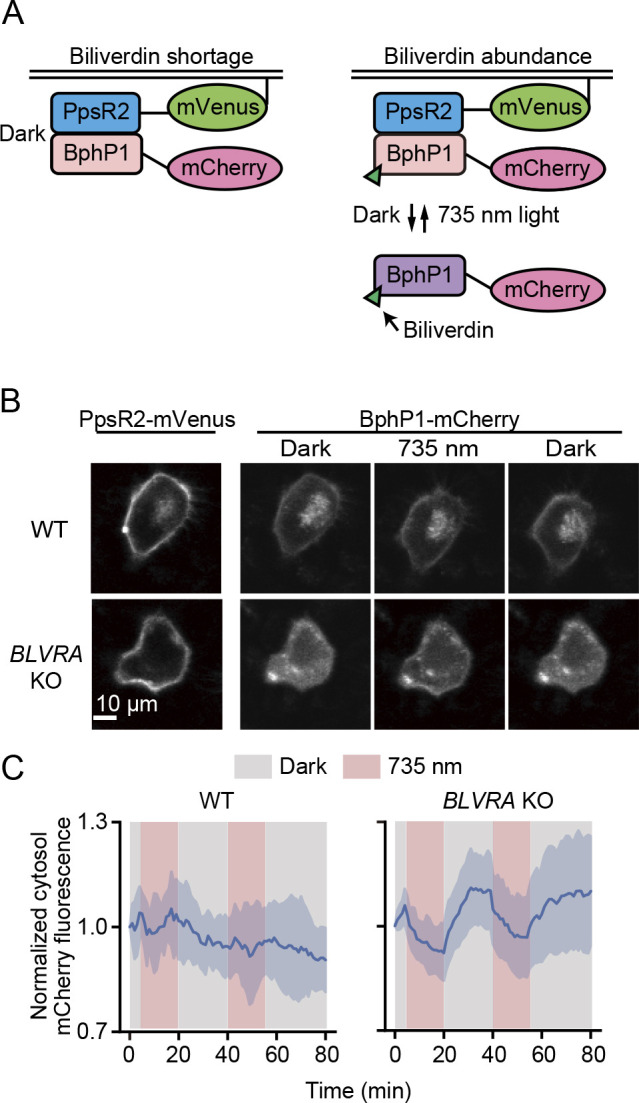
Decreased background signal of the BphP1-PpsR2 system in *BLVRA* KO HeLa cells. (A) Schematic representation of the BphP1-PpsR2 system. Under BV deficiency, BphP1 without BV constitutively binds to PpsR2-mVenus-KRasCAAX at the plasma membrane (left). In the presence of an abundance of BV, BphP1-mCherry binds BV to diffuse into the cytosol in darkness, and moves back to the plasma membrane upon illumination with 735 nm NIR light. (B) Plasma membrane translocation assay in WT and *BLVRA* KO HeLa cells expressing BphP1-mCherry and PpsR2-mVenus-KRasCAAX. HeLa cells were kept under a dark condition for more than 10 min, stimulated by 15 min illumination (735 nm, 14 mW cm^–2^; 735 nm) and recovered in darkness. (C) Time courses of mCherry fluorescence in the cytoplasm. Cells were stimulated with two rounds of illumination (red bands) with a 20 min intermission. The intensity was normalized to that at time zero. The average values (bold curves) are drawn with the SD of 5 cells for WT and 10 cells for *BLVRA* KO.

Finally, we evaluated the applicability of *BLVRA* knockout (KO) to an NIR light-sensing optogenetic tool, the BphP1-PpsR2 optogenetic system (Kaberniuk *et al.*, 2016). The photo-responsive element BphP1 requires BV as a chromophore. BphP1-PpsR2 hetero-dimerization is induced by NIR light, and relieved spontaneously under a dark condition (Fig. 5A). Notably, a shortage of BV is known to yield BV-free BphP1, which binds to PpsR2 regardless of the illumination condition (Fixen *et al.*, 2014), and results in a reduced signal-to-noise ratio. We tested whether *BLVRA* KO enhance the signal-to-noise ratio of the optogenetic tool by using HeLa cells expressing BphP1-mCherry and PpsR2-mVenus-CAAX. In the WT background, BphP1-mCherry was localized at the plasma membrane irrespective of NIR illumination (Fig. 5B and C, Movie 1). In stark contrast, in *BLVRA* KO HeLa cells, BphP1 exhibited light-sensitive shuttling between the cytoplasm and the plasma membrane (Fig. 5B and C, Movie 1). These results demonstrate that removal of BLVRA is an effective strategy when using a BphP1-PpsR2 optogenetic system.

## Discussion

Here we have shown that the removal of BLVRA markedly improves the performance of NIR-FPs and an NIR optogenetic tool in mammalian cells. In the first report on NIR FPs, BV was intravenously injected to increase the fluorescence in mice (Shu *et al.*, 2009). Later, another class of FP, named smURFP, was developed from the allophycocyanin α-subunit, and its fluorescence *in vivo* was also increased markedly by injecting a membrane-permeable BV analog, BVMe_2_ (Rodriguez *et al.*, 2016). These results clearly show that the intracellular concentration of BV is not sufficient to fulfill the potential of NIR-FPs. To genetically increase intracellular BV, HO was overexpressed in *Drosophila*, which increased mIFP fluorescence by 30–40 fold (Yu *et al.*, 2015). In this study, we have shown that *Blvra* knockout is comparable or even better than BV supplementation for the enhancement of iRFPs fluorescence (Fig. 2). In *Blvra^–/–^* mice, the iRFP713 intensity was increased in various organs (Fig. 3B and 3C), demonstrating that *Blvra^–/–^* mice provide an easy and efficient method to expand the possible application of NIR-FPs.

An obvious concern about the use of *Blvra^–/–^* mice is the effect of the removal of BLVRA on the physiological state. There are four reports on the *Blvra* knock-out mouse (Table І). Hinds *et al.* deleted *Blvra* specifically in hepatocytes (Hinds *et al.*, 2006). Although the mutant showed enhanced fasting hyperglycemia and hyperinsulinemia, the knockout did not cause changes in body weight, body composition or plasma bilirubin levels. Two studies used the same mouse line in which exon 3 of *Blvra* was deleted throughout the body: Chen *et al.* reported that the *Blvra^–/–^* mice were born without any gross abnormality difference but exhibited increased oxidization of cholesteryl esters, while Vasavda *et al.* reported that these *Blvra^–/–^* mice were hypersensitive to superoxide. More recently, Bisht *et al.* deleted *Blvra* in myeloid cells and found that C5aR1 and some chemokines including RANTES and IP10 were increased (Bisht *et al.*, 2019). On the other hand, there has been only one report on human *BLVRA* deficiency. Nytofte *et al.* reported on two patients with BLVRA deficiency who did not exhibit any abnormal phenotype other than “green jaundice” (Nytofte *et al.*, 2011). Collectively, these previous results and our own observations in the *Blvra^–/–^* mouse line indicate that BLVRA deficiency does not affect the reproductive rate, weight, growth, or lifespan, but may cause modest anomalies in some signal transduction pathways. Notably, no biosensors or optogenetic tools can avoid the perturbation of cellular signaling pathways. The higher the signals we wish to gain, the larger the perturbation by these tools will become. Use of the *Blvra^–/–^* mice will help researchers to reduce the expression level of biosensors or optogenetic tools. Thus, we should weigh the advantages of the increased signal and the disadvantages of the perturbation of the signaling pathway in *Blvra^–/–^* mice.

Enhancement of the iRFP713 signal varies among tissues (Fig. 3). This difference may have been caused by the tissue-dependent activities of three enzymes, BLVRA, HO, and heme synthesis. Most strikingly, the iRFP713 signal in the brain was barely detectable in the WT mice, but was clearly observed in *Blvra^–/–^* mice (Fig. 3D). BLVRA and HO2 are expressed more abundantly in the brain than in the other organs (Komuro *et al.*, 1996; Vasavda *et al.*, 2019; Zhao *et al.*, 2006). Taking into account the marked enhancement of the iRFP713 signal in *Blvra^–/–^* mice, we speculate that BLVRA activity is higher in the brain than in the other organs. In contrast, iRFP713 intensity of the liver was enhanced only slightly in *Blvra^–/–^* mice (Fig. 3B and 3C). In the liver, despite the high heme content and HO activity (Ingi *et al.*, 1996; Maines, 1988), BLVRA expression level is relatively low compared to other tissues (Komuro *et al.*, 1996; Vasavda *et al.*, 2019), suggesting high levels of BV and BV-binding iRFP713 in the WT mouse liver.

In summary, we generated the *Blvra^–/–^* mouse line, which provides a basis for brighter NIR-FP signals *in vivo mice*, and a promising solution for a larger dynamic range of NIR-optogenetic tools. The *Blvra^–/–^* mouse will be a versatile tool for NIR intravital microscopy.

## Figures and Tables

**Table I:** Phenotype of Blvra-knockout mice

Tissue	Phenotypes	Strain and target	Refs
Liver	Hepatic lipid accumulation under a high fat diet. Enhanced fasting hyperglycemia and hyperinsulinemia.	C57BL/6J Deletion of Exon 2	Hinds *et al.*
Whole body	Enhanced endogenous oxidative stress: higher concentrations of cholesteryl ester hydroperoxides and oxidized peroxiredoxin 2.	C57BL/6J Deletion of Exon 3	Chen *et al.*
Whole body	Enhanced sensitivity to oxidative stress in neurons.	C57BL/6J Deletion of Exon 3	Vasavda *et al.*
Myeloid cells	Enhanced chemokine expression and chemotaxis in myeloid cells.	129S/C57BL/6J Deletion of exons 4 and 5	Bisht *et al.*

## Abbreviations

NIR: near-infrared
BV: biliverdin
BLVR: biliverdin reductase
TPEM: two-photon excitation microscope
BphP: bacterial phytochrome photoreceptors
BVMe_2_: biliverdin dimethyl ester
HO: heme oxygenase
MEFs: mouse embryonic fibroblasts
