# Supplementary figures and images for: Biliverdin Reductase-A Deficiency Brighten and Sensitize Biliverdin-binding Chromoproteins
Source: Cell Struct Funct. 2020 Jun 25;45(2):131–41. doi: 10.1247/csf.20010 (PMC10511041; doi:10.1247/csf.20010)

Figure S1

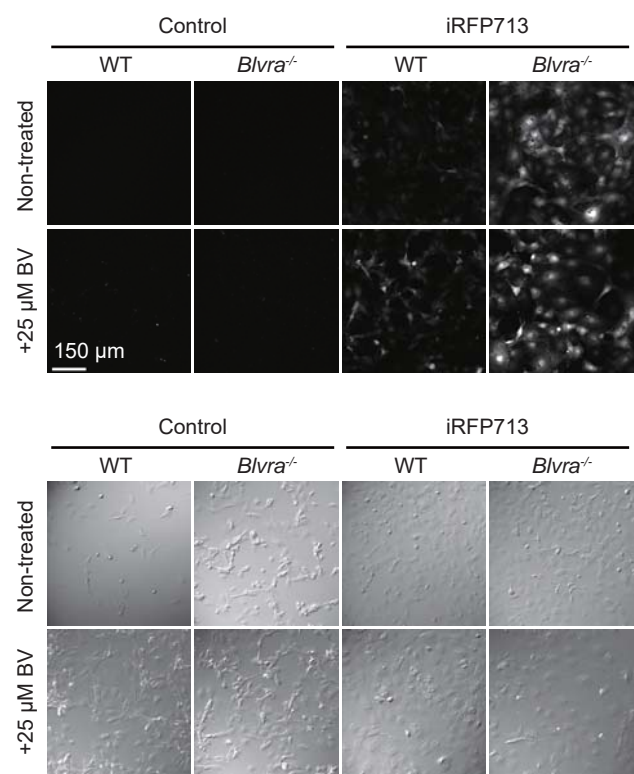

Supplement: Supplementary file 1 — Fig. S1 [file csf_45_20010_1.pdf]

Figure S2

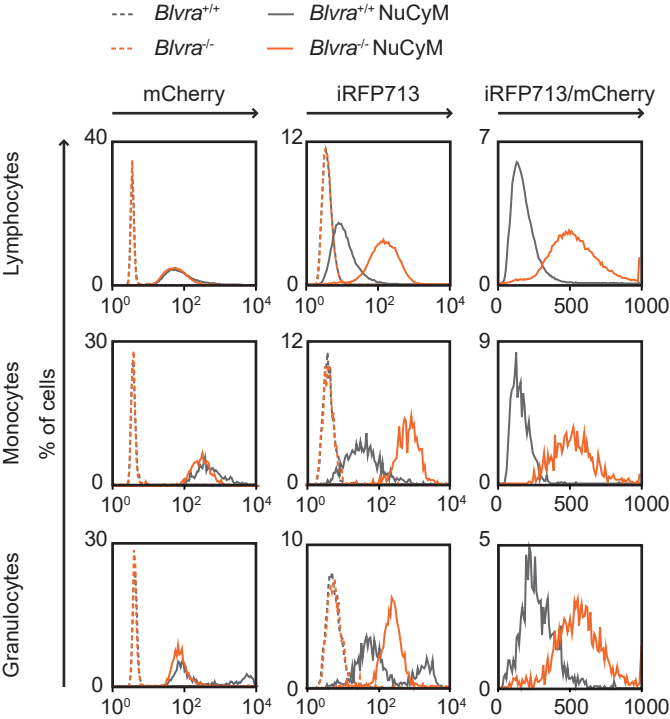

Supplement: Supplementary file 2 — Fig. S2 [file csf_45_20010_2.pdf]
